# How much will older adults exercise? A feasibility study of aerobic training combined with resistance training

**DOI:** 10.1186/s40814-016-0116-5

**Published:** 2017-01-26

**Authors:** Ryan S. Falck, Jennifer C. Davis, Elizabeth Milosevic, Teresa Liu-Ambrose

**Affiliations:** 1 Faculty of Medicine, Aging, Mobility and Cognitive Neuroscience Laboratory, Centre for Hip Health and Mobility, Vancouver Coastal Health Research Institute, Robert H.N. Ho Research Centre, 5th Floor, 2635 Laurel St, Vancouver, BC V5Z 1M9 Canada; 2 Faculty of Medicine, School of Kinesiology, University of British Columbia, 6081 University Blvd, Vancouver, BC V6T 1Z1 Canada; 3 Department of Physical Therapy, Faculty of Medicine, Aging, Mobility and Cognitive Neuroscience Laboratory, Djavad Mowafaghian Centre for Brain Health, University of British Columbia, 212-2177 Wesbrook Mall, Vancouver, BC V6T 1Z3 Canada

**Keywords:** Older adults, Exercise, Aerobic training, Resistance training, Feasibility study

## Abstract

**Background:**

Both aerobic training (AT) and resistance training (RT) have multidimensional health benefits for older adults including increased life expectancy and decreased risk of chronic diseases. However, the volume (i.e., frequency*time) of AT combined with RT in which untrained older adults can feasibly and safely participate remains unclear. Thus, our primary objective was to investigate the feasibility and safety of a high-volume exercise program consisting of twice weekly AT combined with twice weekly RT (i.e., four times weekly exercise) on a group of untrained older adults. In addition, we investigated the effects of the program on physical function, aerobic capacity, muscular strength, and explored factors related to participant adherence.

**Methods:**

We recruited eight inactive older adults (65+ years) to participate in a 6-week, single-group pre-post exercise intervention, consisting of 2 days/week of AT plus 2 days/week of progressive RT for 6 weeks. We recorded program attendance and monitored for adverse events during the course of the program. Participants were tested at both baseline and follow-up on the following: (1) physical function (i.e., timed-up-and-go test (TUG) and short physical performance battery (SPPB)), (2) aerobic capacity (VO_2_max) using the modified Bruce protocol; and (3) muscular strength on the leg press and lat pull-down. Post intervention, we performed qualitative semi-structured interviews of all participants regarding their experiences in the exercise program. We used these responses to examine themes that may affect continued program adherence to a high-volume exercise program.

**Results:**

We recorded an average attendance rate of 83.3% with the lowest attendance for one session being five out of eight participants; no significant adverse events occurred. Significant improvements were observed for SPPB score (1.6; 95% CI: [0.3, 2.9]), VO_2_max (8.8 ml/kg/min; 95% CI: [2.8, 14.8]), and lat pull-down strength (11.8 lbs; 95% CI: [3.3, 20.2]). Qualitative results revealed two themes that promote older adults’ adherence: (1) convenience of the program and (2) the social benefits of exercise.

**Conclusions:**

Our findings suggest untrained older adults can be successful at completing twice weekly AT combined with twice weekly progressive RT; however, these exercise programs should be group-based in order to maintain high adherence.

## Background

Exercise has multidimensional health benefits for older adults including (1) increased life-expectancy [1], (2) reduced risk of chronic diseases such as cardiovascular disease [2], type II diabetes mellitus [3] and dementia [4], and (3) improved mental health and well-being [5]. Regular exercise provides distinct physiological benefits for older adults including improved physical function [6], aerobic capacity [7], muscular strength [8], and cognitive function [9]. As such, the potential benefits of exercise cannot be understated.

For older adults to gain maximum benefit from exercise requires the precise prescription of frequency, intensity, type, and time [10]. Frequency refers to how often the exercise occurs, usually in days per week. Intensity is the level of exertion during the exercise and can be expressed via multiple methods (e.g., Borg rate of perceived exertion [11, 12], heart rate, repetition maximum). Type refers to the modality of exercise, and time refers to the duration of the exercise bout (in minutes). Each of these components is important towards eliciting the dose-response benefits of exercise; but most importantly, each exercise program requires a sufficient—but safe—amount of volume (i.e., frequency*time) and intensity to elicit adaptation [13].

Two different types of exercise training (i.e., aerobic training (AT) and resistance training [RT])—when manipulated through a controlled progression in volume and intensity—consistently demonstrate benefits for healthy aging including cardiovascular health, mobility, and quality of life [14]. AT consists of repetitive movements specifically targeting the cardiovascular system [15]. Strong and accumulating evidence suggests AT is an important contributor to healthy aging and can positively impact older adult cardiovascular health, lipid profile, glucose tolerance, body composition, and bone density [16]. RT consists of muscle-strengthening exercises typically performed with free weights or machines. These exercises may cause positive adaptations in a myriad of factors including (1) markedly increased muscle mass, strength, and power; (2) improved body composition; (3) mobility and balance; and (4) improved quality of life [17, 18]. As such, current recommendations suggest older adults regularly engage in (1) moderate intensity AT 5 days/week for at least 30 min/session or 3 days/week of vigorous intensity for at least 20 min/session; (2) moderate intensity RT at least twice per week; and (3) participating in more AT and RT should be encouraged [14]. While the current ACSM guidelines are indeed a worthwhile goal, there are no specific guidelines for untrained older adults (i.e., have not previously engaged in exercise training in the past 6 months) regarding the maximum total volume of exercise training that is a safe and feasible starting point. Thus, an important next step is to determine whether untrained older adults who are initiating an exercise program can tolerate high volumes of exercise training.

Another essential component of establishing an effective exercise program is understanding the factors that motivate adherence. While current guidelines suggest older adults should engage in regular physical activity of at least 150 min per week [14], most older adults are unable to meet these recommendations [19]. The most common barriers to meeting these guidelines include (1) poor health, (2) environmental barriers, and (3) a lack of knowledge of how to safely participate in physical activity or an exercise program [20]. These barriers can also significantly affect long-term exercise adherence [21] and thus prevent older adults from experiencing the long term benefits of exercise training. As such, identifying factors for what makes older adults more likely to engage in—and adhere to—regular exercise training is an important line of inquiry.

The safety and feasibility of older adults engaging in both AT and RT concurrently is also not well established. Previous research suggests older adults can safely complete a 3 days/week exercise program consisting of AT and RT in each exercise session [22] and 4 days/week of AT alone [23]. While this preliminary evidence is promising, it is still unknown what the underlying factors of high-volume exercise programs are which make older adults more likely to adhere [24]. Thus, research illustrating the experiences of older adults at exercise volumes greater than 3 days/week is needed. This qualitative information could shed light on ways to make higher volumes of exercise feasible and enjoyable for older adults.

Hence, as an important next step, we examined the feasibility and safety of a 6-week exercise program for adults ≥65 years of age consisting of twice weekly AT combined with twice weekly RT. We also investigated if this exercise program would lead to improvements in (1) physical function, (2) aerobic capacity, and (3) muscle strength. Finally, we examined the experiences of older adults engaged in high-volume exercise and the factors related to program adherence.

## Methods

This study was designed as a single group pre-post exercise intervention. This study was approved by Vancouver Coastal Health Research Institute and the University of British Columbia’s Clinical Research Ethics Board (H15-02181). All participants provided written informed consent.

### Participants

We recruited eight community dwelling older adults (65+ years) from December 1, 2015–January 9, 2016, via advertisement at both University of British Columbia and Vancouver General Hospital, British Columbia, Canada. Interested individuals contacted the study coordinator via telephone. We then performed a brief telephone screening to determine study eligibility, and individuals who appeared to meet study criteria were invited to an information session.

Participants were eligible if they (1) were between 65–80 years of age, (2) were not regularly exercising for the past 6 months (>60 min/week), (3) did not have any significant musculoskeletal issues, (4) were not diagnosed with peripheral neuropathy, (5) did not have a high risk for cardiac complications during exercise, (6) had not previously suffered a stroke, (7) had no moderate-to-severe respiratory diseases, (8) score >24/30 on the mini-mental state exam (MMSE; [25]), and (9) did not have any other medical condition precluding them from exercise.

We provided potential participants who attended the information session with details of the study and allowed them to ask questions. Study personnel scheduled consent and screening sessions for interested participants. Those who remained eligible were scheduled for baseline assessment.

### Measures

Trained staff members administered all testing procedures. A trained exercise physiologist was present for all exercise testing sessions.

#### Demographics

At study entry, we obtained general health history and demographic information by questionnaire. In addition, we ascertained height and body weight using a calibrated stadiometer and a balance-beam scale, respectively. Height and weight were used to determine body mass index (BMI; kg/m^2^).

#### Feasibility and safety

To determine the feasibility of the program, we collected attendance records from each AT and RT session. We considered the exercise program to be feasible if we maintained >50% attendance for all sessions and averaged >80% attendance per session. In addition, we monitored and recorded adverse events during the course of the study to determine the safety of the program.

#### Physical function

We assessed physical function of the participants using the timed-up-and-go test (TUG) [26] and the short physical performance battery (SPPB) [27]. For the TUG, participants rose from a standard chair, walked a distance of three meters, turned, walked back to the chair, and sat down [26]. We recorded the time in seconds to complete the TUG, based on the average of two separate trials. For the SPPB, participants were assessed on performances of standing balance, walking (4 m), and sit-to-stand. Each component is rated out of 4 points, for a maximum of 12 points; a score <9 predicts subsequent disability [27].

#### Aerobic capacity

We measured maximal aerobic capacity (VO_2_max) using the modified Bruce protocol [28], which is a submaximal graded exercise test. The modified Bruce protocol is frequently used to estimate VO_2_max in older populations, due to the reduced level of stress it places upon the participant [28]. We monitored participant blood pressure and heart rate throughout the treadmill test according to standard procedures and terminated the test when the participant reached volitional fatigue. VO_2_max was calculated using the following formula as recommended by Bruce and colleagues [29]:

$$ \mathrm{Estimated}\ {\mathrm{VO}}_2 max = 6.70\ \hbox{--}\ 2.82*\mathrm{Sex} + 0.056*\mathrm{Time} $$

where the weighting factor is 1 = females and 2 = males and time is recorded in seconds.

#### Muscular strength

We assessed muscular strength for all RT machines on Keiser pressurized air resistance machines (Keiser Corporation: Fresno, CA) using the 10-repetition maximum (10-RM) test. We measured muscular strength during the second week of the exercise program in order to allow participants to become acclimated with using the RT machines properly and to avoid excessive risk of injury during 10-RM testing. At follow-up testing, leg press, and lat pull-down were retested to determine upper- and lower-body strength gains.

The 10-RM is generally used for older adult populations due to the reduced absolute intensity of the exercise, as well as for increased safety [30–32]. We estimated initial loads for the 10-RM test based on researcher experience and feedback from verbal questions pertaining to training history. If the participant reached 12 repetitions, and reported being able to do more, then the participant was given a 5-min rest period followed by a subsequent 10-RM test wherein we increased the resistance by approximately 20%. This process was repeated until the participant reached fatigue within 10 repetitions. We then estimated 1-RM for the strength exercises according to established guidelines [33]. These estimations were used to calculate the RT intensity for the remainder of the program, with the target of training being 60–65% of 1-RM.

### Procedure

Following baseline testing, participants underwent 6 weeks of four times weekly exercise training. Each session was approximately 60 min and included (1) a 10-minute warm up, (2) 40 min of training (AT or RT), and (3) a 10-min cool down. We kept attendance records for all sessions, which were led by a trained exercise physiologist and assisted by multiple staff members. We monitored the intensity of each training session using the 20-point Borg rating of perceived exertion (RPE) [11, 12].

#### AT program

Table 1 details the AT protocol. The AT twice-weekly program followed a previously used protocol for older adults [34, 35]. All sessions involved walking outside at an age-specific heart rate reserve (HRR) which gradually progressed from 45% HRR in the first week to 60% HRR in the final week of the program. Participants wore heart rate monitors at each AT session and were instructed to maintain a target heart rate based on the %HRR. Once a participant reached their prescribed target heart rate, they were instructed to maintain the heart rate for the remainder of the session. The staff monitored the participants at 15-min intervals for their heart rate and RPE.

**Table 1:** Aerobic training progression

		Week 1		Week 2		Week 3		Week 4		Week 5		Week 6	
	Exercise	Time	%HRR^a^	Time	%HRR	Time	%HRR	Time	%HRR	Time	%HRR	Time	%HRR
Aerobic training day 1	Warm up	10 min	35%	10 min	35%	10 min	40%	10 min	40%	10 min	40%	10 min	40%
	Training	40 min	45%	40 min	50%	40 min	55%	40 min	55%	40 min	60%	40 min	60%
	Cool down	10 min	35%	10 min	35%	10 min	40%	10 min	40%	10 min	40%	10 min	40%
		Time	%HRR	Time	%HRR	Time	%HRR	Time	%HRR	Time	%HRR	Time	%HRR
Aerobic training day 2	Warm up	10 min	35%	10 min	35%	10 min	40%	10 min	40%	10 min	40%	10 min	40%
	Training	40 min	45%	40 min	50%	40 min	55%	40 min	55%	40 min	60%	40 min	60%
	Cool down	10 min	35%	10 min	35%	10 min	40%	10 min	40%	10 min	40%	10 min	40%

^a^%HRR: estimated %heart rate reserve

#### RT program

Table 2 details the twice-weekly RT protocol. During the first week of training, we instructed participants on the use of the six RT machines. During the second session of RT in the second week, participants tested for 10-RM strength on all exercises. In all subsequent weeks of training, participants performed the exercises using weight prescribed based off of estimations of %1-RM. The program leader increased the resistance if the exercise appeared very easy for the participant or if the participant felt the intensity of the exercise was light (i.e., RPE <10).

**Table 2:** Resistance training progression

	Exercise	Week 1		Week 2		Week 3		Week 4		Week 5		Week 6	
Resistance training day 1		Set 1	Set 2	Set 1	Set 2	Set 1	Set 2	Set 1	Set 2	Set 1	Set 2	Set 1	Set 2
		Reps	Reps	Reps	Reps	%RM × Reps	%RM × Reps	%RM × Reps	%RM × Reps	%RM × Reps	%RM × Reps	%RM × Reps	%RM × Reps
	Leg press	12	–	12	12	60% × 12	60% × 12	60% × 12	60% × 12	65% × 10	65% × 10	65% × 12	65% × 12
	Back row	12	–	12	12	60% × 12	60% × 12	60% × 12	60% × 12	65% × 10	65% × 10	65% × 12	65% × 12
	Hamstring curl	12	–	12	12	60% × 12	60% × 12	60% × 12	60% × 12	65% × 10	65% × 10	65% × 12	65% × 12
	Lat pull-down	12	–	12	12	60% × 12	60% × 12	60% × 12	60% × 12	65% × 10	65% × 10	65% × 12	65% × 12
	Bicep curl	12	–	12	12	60% × 12	60% × 12	60% × 12	60% × 12	65% × 10	65% × 10	65% × 12	65% × 12
	Triceps extension	12	–	12	12	60% × 12	60% × 12	60% × 12	60% × 12	65% × 10	65% × 10	65% × 12	65% × 12
	Wall pushups	–	–	–	–	–	–	8	8	10	10	10	10
	Body weight squats	–	–	–	–	–	–	8	8	10	10	10	10
Resistance training day 2		Set 1	Set 2	Set 1	Set 2	Set 1	Set 2	Set 1	Set 2	Set 1	Set 2	Set 1	Set 2
		Reps	Reps	Reps	Reps	%RM × Reps	%RM × Reps	%RM × Reps	%RM × Reps	%RM × Reps	%RM × Reps	%RM × Reps	%RM × Reps
	Leg press	15	–	10	10-RM test	60% × 10	60% × 10	60% × 10	60% × 10	65% × 8	65% × 8	65% × 10	65% × 10
	Back row	15	−	10	10-RM test	60% × 10	60% × 10	60% × 10	60% × 10	65% × 8	65% × 8	65% × 10	65% × 10
	Hamstring curl	15	–	10	10-RM test	60%×10	60%×10	60%×10	60%×10	65%×8	65%×8	65%×10	65%×10
	Lat pull-down	15	–	10	10-RM test	60% × 10	60% × 10	60% × 10	60% × 10	65% × 8	65% × 8	65% × 10	65% × 10
	Bicep curl	15	–	10	10-RM test	60% × 10	60% × 10	60% × 10	60% × 10	65% × 8	65% × 8	65% × 10	65% × 10
	Triceps extension	15	–	10	10-RM test	60% × 10	60% × 10	60% × 10	60% × 10	65% × 8	65% × 8	65% × 10	65% × 10
	Wall pushups	–	–	–	–	–	–	8	8	10	10	8	8
	Body weight squats	–	–	–	–	–	–	8	8	10	10	8	8

*Note:* During week 1 and week 2 of RT, participants were instructed to choose “easy and comfortable” weights to use for each RT exercise. A 10-repetition maximum (10-RM) test was performed at the end of week 2. *%R*M: estimated % of 1-repetition max

### In-depth interview at study completion

We conducted in-depth, open-ended follow-up interviews with all eight participants in order to understand their experiences of the program. We conducted these semi-structured interviews via an interpretivist perspective in order to understand the complex world of lived experience [36]. These interviews lasted approximately 20 min and took place at the Centre for Hip Health and Mobility at Vancouver General Hospital.

We used nine open-ended questions in each interview, designed to elicit responses about program satisfaction and potential areas for improvement. Participants were made aware that the purpose of the interview was to better understand their experiences during the exercise program. Questions examined the following: (1) overall program experience, (2) most enjoyable and least enjoyable aspects of the program, (3) easiest and most difficult aspects of the program, (4) subjective improvements in quality of life, (5) areas of the program participants would change, (6) thoughts on committing to a six month exercise program, (7) likelihood to continue this exercise program longer than 6 months, (8) thoughts on the cost and benefit of a four times per week exercise program, and (9) preferences for either AT or RT. When warranted, the interviewer used prompters to stimulate further elucidation of ideas. The same trained female author (EM) conducted, recorded, and transcribed all interviews to ensure consistency across the data set.

### Data analysis

We scored all measures according to standard procedures and assessed whether changes in outcome measures differed from zero using a one-sample *t* test. For each outcome measure, change was calculated as follow-up minus baseline. We present 95% CIs for each of the statistical tests performed. We also calculated the observed effect size (Cohen’s *d*) for each outcome of interest using the following formula: (mean post test − mean baseline)/(baseline standard deviation). Because of our small sample size, these calculations helped to determine the potential clinical meaningfulness of the results in addition to statistical significance. Conventions of small (*d* = 0.20), medium (*d* = 0.50), and large (*d* = 0.80) were used [37]. The results of these hypothesis tests and effect sizes should be treated with caution and as preliminary, given our small sample size.

We analyzed the follow-up interview transcripts according to the three stages of qualitative analysis outlined by Carpenter and Suto [36]: data reduction, data display, and conclusion drawing/verification. Briefly, in the initial data reduction stage, two authors (RSF and EM) repeatedly read the transcripts in order to highlight sections of data that informed the research question. We then assigned codes to data fragments with similar meanings and overlapping codes were then grouped into categories. In the subsequent phase of analysis, these categories of data were refined and clustered together to form preliminary themes by two of the authors (RSF and EM). The tentative themes were then summarized in a table to provide a visual representation of the data, thus facilitating further examination of patterns, relationships, and meanings. Lastly, in the conclusion drawing and verification stage, two of the authors established a finalized set of overarching themes (RSF and EM).

## Results

### Participant characteristics

Participant characteristics are described in Table 3. The majority of the participants were female (75.0%) and non-smokers (87.5%) and had a mean age of 73.6 ± 1.6 years. Mean BMI was 24.5 ± 5.3 kg/m^2^. The most common co-morbidity was osteoporosis (25.0%).

**Table 3:** Participant characteristics (*N* = 8)

Participant characteristic	Mean (SD) or frequency (%)
Females	87.5%
Age	73.4 (1.6)
BMI (kg/m^2^)	24.1 (5.6)
Comorbidities	
Osteoporosis	25.0%
Skin cancer	12.5%
Type 2 diabetes	12.5%
Smoking status	
Never smoked	87.5%
Former smoker	12.5%
Baseline physical performance	
Timed up-and-go (s)	7.1 (3.4)
SPPB^a^ score	10.6 (1.4)
Estimated 1-RM leg press	388.2 (277.8)
Estimated 1-RM lat pull-down	94.5 (48.7)
VO_2_max (ml/kg/min)^b^	39.4 (8.6)

^a^Short physical performance battery

^b^Maximal aerobic capacity measured by modified Bruce protocol

### Feasibility and safety

**Fig. 1:**
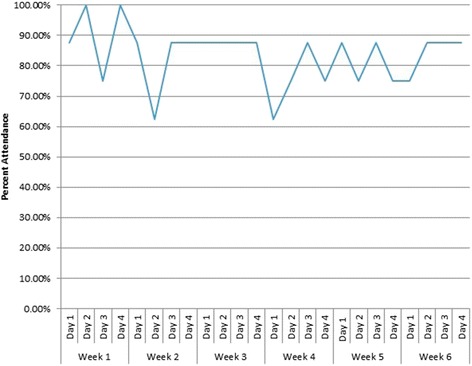
Attendance averaged 83.33% per session with two sessions having the lowest attendance of 62.50%

Participant attendance is described in Fig. 1. Participant attendance averaged 83.3% per session. The lowest attendance per session was five participants (i.e., 62.5%), which occurred twice. Two individuals had 100.0% attendance. We observed no significant adverse events during the course of this study.

### Changes in outcome measures

Changes in outcome measures are summarized in Table 4. We calculated a moderate effect size improvement for TUG (*d* = −0.34), although we did not find a significant improvement after 6 weeks. There was a significant improvement in SPPB score following the program (1.6; 95% CI: [0.3, 2.9], *p* = 0.02) and found to be a large effect size (*d* = 1.12). Estimated lat pull-down 1-RM improved significantly (11.8 lbs; 95% CI: [3.3, 20.2], *p* = 0.02) with a large effect size (*d* = 1.46). We also found a trend towards significant improvement in estimated leg press 1-RM (97.1 lbs; 95% CI: [−8.1, 202.3], *p* = 0.06) with a large effect size (*d* = 0.97). We found a significant improvement in VO_2_max (8.8 ml/kg/min; 95% CI: [2.8, 14.8], *p* = 0.01) with a large effect size (*d* = 1.36).

**Table 4:** Changes in outcomes and effect sizes

Outcome	Mean (95% CI)	*t* value	*p* value	Cohen’s *d*
∆Timed up-and-go (s)	−1.5 (−5.0, 2.9)	−0.96	0.37	−0.34
∆SPPB score^a^	1.6 (0.3, 2.9)	2.98	0.02	1.12
∆Estimated 1-RM^b^ leg press (lbs.)	97.1 (−8.1, 202.3)	2.37	0.06	0.97
∆Estimated 1-RM lat pull-down (lbs.)	11.8 (3.3, 20.2)	3.58	0.02	1.46
∆VO_2_max (ml/kg/min)^c^	8.8 (2.8, 14.8)	3.60	0.01	1.36

^a^Short physical performance battery

^b^Repetition maximum

^c^Maximal aerobic capacity measured by modified Bruce protocol

### Responses to follow-up interviews

We identified two parent themes: (1) factors impacting program satisfaction (Table 5) and (2) barriers for continued participation (Table 6). Factors influencing program satisfaction included (1) program convenience, (2) program novelty, and (3) social benefits of exercise. Barriers for continued participation included long wait times during the RT sessions and other commitments outside of the exercise program.

**Table 5:** Factors influencing program satisfaction

Program convenience
• “My kids want me to join [a] seniors exercise group, but then parking is very expensive there…I can just walk here.”
• “Ten in the morning is perfect for exercise and walking. It’s only an hour and it’s in the morning, and then you’ve done something good and have the rest of the day”
Program novelty
• “My favorite part was going down [to the water] where I haven’t walked before. It was so beautiful and it was a totally new experience because I had never walked down there.”
• “A first I was anxious because of the machines that we will be using… Yeah, I said ‘mhm I can’t remember the name of what we’re doing’ so I said ‘can I get pictures of these machines?’ because my kids will ask me what am I doing or what are the things that I use and I can’t even [tell them].”
Social benefits of exercise
• “I enjoyed the people here; they were all great – very helpful every one of them… Motivation is if you have somebody, I find myself personally, that helps a lot.”
• “I joined the [gym] for a while, well for one month. I went three times and all three times when I asked the girl there at the desk if she would show me how certain machines work, every time, all three times, she said ‘I’m just going on my break in a few minutes’. Yeah, and she’s the only one on the desk, you know that was there to help. Yeah, and it wasn’t a very friendly place anyway. There were just people doing their own thing and yeah… so I cancelled my membership in one month. Yeah I’m not going to put myself through a thing where I’m unhappy because I don’t know what to do and how to do it. When you’re a senior that kind of thing is kind of devastating – when you don’t know how a machine works and you want to get fit, you know.”
• “I enjoyed also the group, the very small group, and it’s good that you get to know these people. And talking while walking is really good too… I didn’t feel tired because I was walking and talking at the same time, so I really enjoyed that.”
• “I liked the walking I guess…because it was outdoors and it was spring and I could see life coming, the return of life.”

**Table 6:** Barriers to continued participation

Long wait times during RT sessions
• “When we do the weights that time, a little bit we had to wait, a little bit down time. But I don’t think there’s another way to do it. Unless we were trained and we were told what’s the program we can do on our own, and you don’t have to wait, have the down time. Sometimes it feels a little bit long, a little bit boring. But in general I think it’s okay. But if you are in the group and somebody is really slow they drag the whole group.”
• “What I thought would have helped is if we had been grouped according to our capability. Because some of us, we’re not the same size and we’re not the same age and some people were faster… you [wouldn’t] have to change the settings as much.”
Other activity commitments
• “Yeah I probably could [continue to participate]. As long as it wasn’t in the summer because in May, June, July, August, and September I lawn bowl…and I’m getting quite good at it so I go on the odd tournament now. So that kind of takes up some of my time.”
• “In fact, I don’t mind to participate. And to be honest, I like to take part of it, but I have to know ahead of time because I don’t want to quit in the middle of the program. If I commit to something, I rather finish, to complete it, right.”

#### Factors impacting program satisfaction

Participants were more likely to enjoy the program if they found the scheduling of classes and location to be convenient. Several participants noted that they enjoyed the class more because it was early in the morning, and it was close to where they lived. The novelty of the training program also directly related to participant satisfaction. For example, participants enjoyed that AT was a new experience because they had never been to several of the walking locations. Several participants also noted the outdoor setting of the AT sessions as a contributing factor to their satisfaction with the program. Participants described how being outdoors contributed to their enjoyment of exercise.

Another factor, which impacted participant satisfaction, was the social environment of the classes. Participants thought the instructors created a welcoming environment by addressing them using their names and being encouraging during the exercise sessions. This environment gave participants more motivation during the exercise sessions. Several participants had access to exercise facilities for independent use; however, they often expressed apprehension about exercising on their own. Participants described the atmosphere of these facilities as “unfriendly” and thus did not make them feel like exercising regularly. By comparison, our exercise program allowed participants to form new friendships, which made exercise more enjoyable. Social engagement with other participants was also cited as a contributing factor to overall satisfaction.

#### Barriers to continued participation

When asked about their preference between AT and RT, the participants preferred AT. While the reasons for this preference were not always explicitly stated, several participants later discussed how they were unfamiliar with or nervous about using machines. In addition, participants often had to wait for others to complete a station. These wait times sometimes made RT tedious and slow. Participants discussed the possibility of breaking the class into smaller groups according to their speed or overall ability. Dividing the RT sessions into smaller groups of individuals at a similar level of fitness may have improved participant enjoyment.

When asked about participating in a longer exercise program, many participants expressed a desire to do so; however, it was clear each individual had other priorities. Some participants felt continued involvement in a longer exercise program was possible, but they needed to make sure they had the time to commit to the program.

## Discussion

We found that older adults can feasibly and safely participate in a high-volume exercise program consisting of both AT and RT. The strong adherence to our 6-week high-volume exercise program also resulted in significant improvements in physical function, VO_2_max, and muscular strength. Finally, our qualitative results provide evidence that the social environment and the convenience of the program were strongly related to adherence.

Our participants were able to safely complete and adhere to a high-volume exercise program for 6 weeks, with an average attendance rate of 83.3% per session. Moreover, the quantitative results of our study show large improvements in physical function, aerobic capacity, and muscular strength following just 6 weeks of training; however, these results should be treated with caution and as preliminary, given the small sample size. Other studies have found similarly large improvements in each of these domains following exercise training. Taaffe and colleagues [38] found large-scale improvements in physical function and muscular strength of older adults from RT as little as once a week, although older adults were trained for 24 weeks in this study. Cress and others [39] found a 6-month combined AT and RT program had large-scale improvements in physical function, VO_2_max, and muscular strength. However, our results indicate large improvements in both VO_2_max and muscle strength can occur from a much more abbreviated training period via high-volume exercise. Thus, older adults engaged in a high-volume exercise program may be able to more rapidly achieve the benefits of exercise training.

The results of our semi-structured interviews also illustrate that program convenience, the physical and social environment, and the lack of self-efficacy with RT all influenced continued adherence to our high-volume exercise program. Participants found that the accessibility of the program influenced their continued adherence. Previous research has shown accessibility of an exercise program is directly related to exercise adherence in older adults [20, 40]. Our qualitative results also suggested the social benefits of the exercise program were critical in keeping people engaged in the program. Participants consistently mentioned the program gave them an opportunity to make new friends and have new experiences, which helped keep the program interesting. Social engagement is an important part of exercise for older adults [20, 24, 40], and thus, our findings further implicate social engagement as an important factor towards exercise adherence. Of final note, our data suggest AT is preferred over RT in older adults. Walking was a highly familiar activity to the participants whereas RT was often new, unfamiliar, and even initially intimidating. Potentially, this lack of self-efficacy for RT could be eventually overcome in a longer program [41]; however, this is beyond the scope of this investigation. Another likely reason for the general preference for AT over RT was the discomfort caused by progressive RT for untrained older adults [42], and thus, strategies to improve older adult enjoyment of RT are needed.

### Limitations

The exercise program we used was only 6 weeks in duration and thus may not have been long enough to elicit significant adaptations in some of our measures. Our exercise program only examined moderate-intensity exercise (i.e., AT: 45–60% HRR; RT: 60–70% 1-RM) and thus cannot determine whether older adults will safely and feasibly complete a high-intensity exercise program. The moderate-intensity of our exercise program was based on current guidelines for older adults [14], which may be a more feasible starting point for an exercise program than high-intensity exercise. Thus, future research is needed on whether older adults can engage in a high-volume, high-intensity exercise program. A final limitation is our results are only applicable to the training program currently used and could be potentially much different had another frequency and/or intensity of training been used.

## Conclusions

Our study suggests healthy older adults can successfully complete a 6-week high-volume, moderate-intensity, multimodal exercise trial. Our study also provides useful insights into designing future high-volume exercise interventions. Specifically, our qualitative data suggest that the following may promote greater adherence: (1) small groups for RT, (2) if incorporating AT into the program, performing the exercise outside, and (3) scheduling classes early in the day in order to maintain high adherence to a high-volume exercise program.

## Abbreviations

AT: Aerobic training
BMI: Body mass index
HRR: Heart rate reserve
MMSE: Mini-mental state exam
RPE: Rate of perceived exertion
RT: Resistance training
SPPB: Short physical performance battery
TUG: Timed up-and-go test
VO_2_max: Maximal aerobic capacity 10-RM: 10 repetition maximum.
